# The effects of iloprost and beta3 receptor agonist on TRPA1 and TRPC1 immunreactivity in an experimental lower extremty ischemia-reperfusion injury model

**DOI:** 10.3906/sag-2104-68

**Published:** 2021-10-21

**Authors:** Latif ÜSTÜNEL, İbrahim Murat ÖZGÜLER

**Affiliations:** 1 Department of Cardiovascular Surgery, Faculty of Medicine, Fırat University, Elazığ Turkey

**Keywords:** Rat, iloprost, BRL, TRP, ischemia, reperfusion

## Abstract

**Background/aim:**

In this study, we aimed to investigate the effects of antioxidant iloprost (ILO) and ß3 adrenergic receptor agonist (BRL) on transient receptor potential ankyrin 1 (TRPA1) and transient receptor potential canonical 1 (TRPC1) ion channels on an experimental ischemia and reperfusion injury model in 30 male Wistar albino rats aged 8–10 weeks.

**Materials and methods:**

Wistar Albino rats aged were divided into 5 equal groups. Group I Sham operation, Group II IR (ischemia-reperfusion) procedure, Group III IR + intravenous ILO administration, Group IV IR + intraperitoneal BRL administration, and Group V IR + intravenous ILO + intraperitoneal BRL administration group. Two ng/kg/min ILO intravenous infusion was applied to the ILO group. A single dose of 5 mcg/kg BRL intraperitoneal was applied to BRL group. TOS (total oxidant status), TRPA1, and TRPC1 levels were measured with ELISA (enzyme linked immunosorbent assay) in serum, immunohistochemical staining in musculus quadriceps femoris tissue.

**Results:**

Compared with the sham group, the IR group had a statistically significant increase in serum levels of TOS (p = 0.004), TRPA1 (p = 0.002), and TRPC1 (p = 0.008) along with TRPA1- and TRPC1-immunoreactivity (p = 0.005, each) in the tissue. When compared with the IR group in terms of serum levels of TRPA1 and tissue TRPA1-immunoreactivity, although there was no statistically significant difference in the IR+Ilo (p = 0.257 and p = 0.429, respectively), IR+Brl (p = 0.024 and p = 0.177, respectively), and IR+Ilo+Brl (p = 0.024 and p = 0.329, respectively) groups, serum levels of TOS and TRPC1 along with tissue TRPC1-immunoreactivity were statistically significantly reduced in the IR+Ilo (p = 0.002, p = 0.008, and p = 0.004, respectively), IR+Brl (p = 0.004, p = 0.008, and p = 0.004, respectively), and IR+Ilo+Brl groups (p = 0.002, p = 0.008, and p = 0.004, respectively).

**Conclusion:**

In IR group serum TOS, TRPA1 and TRPC1 levels ,and tissue TRPA1 and TRPC1 immunoreactivity were statistically significant increase when compared to the sham group. In IR+ILO, IR+BRL and IR+ILO+BRL groups serum TRPA1 and tissue TRPA1 immunoreactivity did not change when compared to IR group. Serum TOS and TRPC1 levels, tissue TRPC1 immunoreactivty were statistically significant decreased when compared to IR group. More detailed and expanded population studies are needed to discuss our results.

## 1. Introduction

Ischemia reperfusion (IR) injuries are frequently encountered in prolonged lower extremity tourniquets or after reperfusion surgeries following acute trauma. IR damage occurs as a result of the accelerated release of ROS (reactive oxygen species) into circulation and excessive release of inflammatory cytokines [1]. ROS are generated primarily in mitochondria where small amounts of monovalent oxygen are reduced to free radicals through the activation of redox reactions catalyzed by special enzymes of the electron transport chain [2].

Iloprost (ILO) is a PGI2 (prostacyclin) analog [3]. ILO has effects such as inhibition of platelet aggregation, arteriovenous dilatation, increased capillary permeability due to the release of mediators (e.g., serotonin and histamine), increased endogenous fibrinolytic activity, and antiinflammatory effects (such as inhibition of leukocyte adhesion after endothelial damage and reduced TNF (tumor necrosis factor) alpha release [3,4]. BRL 37344 (ß3 adrenergic receptor activation) is a selective ß_3_-receptor agonist (ß3-RA). BRL 37344 shows a cardioprotective effect accompanied by a negative inotropic effect in case BRL catecholamine levels increase [5]. It has also been stated that BRL can be used as an antioxidant in cancer treatment [2].

Transient receptor potential channels are ion channel family consisting of many diverse members [6]. This family is divided into subgroups such as canonical TRP (transient receptor potential), TRPC (canonical TRP), TRPV (vanilloid TRP), TRPM (melastatin TRP), TRPA (ankyrin TRP), TRPML (mucolipin TRP), and TRPP (polycystic TRP). TRP channels have varying degrees of selectivity and permeability to calcium ions. TRPC1 is the first member of the TRPC subgroup found in the tissues of the liver, kidney, testes, ovary, and brain. In addition, TRPC1 is the first cloned TRP protein in mammals and is a founding member of TRPCs (TRPC subgroups) [7]. TRPCs are localized on the plasma membrane and are activated in a G protein-bound receptor-phospholipase C-dependent manner. TRPCs can also be stimulated via receptor tyrosine kinases or lysophospholipids, hypoosmotic solutions, and mechanical stimulants [8]. TRPA1 was discovered by Jaquemar et al. as an ankyrin-like transmembrane protein [9]. It is considered as a separate subgroup of TRP channels due to the presence of ankyrin repeats in its N-terminal region. TRPA1 is a well-defined chemonociceptor that is an ideal target for analgesics, and it is sensitive to cold [10]. TRPA1 is expressed in both peptidergic and nonpeptidergic neurons. Additionally, epithelial cells, melanocytes, mast cells, and fibroblasts can synthesize TRPA1. [11]. The molecular mechanisms of TRPA1 and structure and channel activation of TRPC1 have not yet been fully elucidated [6,7]. In this study, we examined the changes in TRPC1 and TRPA1 during IR damage. We also investigated the effects of ILO and BRL37344 on these changes, and the role of these ion channels in IR damage.

## 2. Materials and methods

This study was conducted between February 2020- January 2021 with the approval from Animal Research Ethics Committee of Fırat University dated 15.01.2020 and numbered 2020/01. The research was performed in University Experimental Research Center Unit between February 2020 and January 2021 after ethical committee approval.

The rats used in our study were kept at a temperature of 22–25 ºC for 12 h of light cycle (7:00–19:00) and 12 h of dark cycle (19:00–7:00). They were fed in special cages whose bottoms were cleaned every day. Feed was given in steel containers and water in glass bottles (tap water). All rats were fed with the same standard feed. Water and feed were provided ad libitum. The rats were housed by daily cleaning of the cages. Thirty male Wistar albino rats aged 8–10 weeks were divided into 5 groups:

**Group I (sham group) (n = 6):** Laparotomy and abdominal aortic dissection were performed on the rats in this group. The stress and duration of the surgical procedure were equal for all the 5 groups.

**Group II (IR group) (n = 6):** In this group, the rats were immobilized in the supine position and laparotomy was performed in the midline of the abdomen to dissect the IAA (infrarenal abdominal aorta). Later, IAA was clipped with a nontraumatic microvascular clamp for 120 min. The clips were then removed, and the surgical procedure was completed after 120 min of reperfusion.

**Group III (IR + ILO group) (n = 6):** Rats were immobilized in the supine position and laparotomy was performed in the midline of the abdomen to dissect IAA. Later, IAA was clipped with a nontraumatic microvascular clamp for 120 min, and 2 ng/kg/min ILO infusion was administered by the jugular vein after the clips were removed. The surgical procedure was completed after 120 min of reperfusion.

**Group IV (IR + BRL group) (n = 6):** The rats in this group were immobilized in the supine position and laparotomy was performed in the midline of the abdomen to dissect IAA. Subsequently, IAA was clipped with a nontraumatic microvascular clamp for 120 min. After the clips were removed, a single dose of 5 mcg/kg ß3 BRL was administered by intraperitoneally. The surgical procedure was completed after 120 min of reperfusion.

**Group V (IR + ILO + BRL group) (n = 6): **The rats in this group were immobilized in the supine position and laparotomy was performed in the midline of the abdomen to dissect IAA. Later, IAA was clipped with a nontraumatic microvascular clamp for 120 mins. After the clips were removed, 2 ng/kg/min ILO infusion was administered by the jugular vein with a single dose of 5 mcg/kg BRL by intraperitoneally after 120 min of declamping the IAA (infrarenal abdominal aorta) the surgical procedure was completed.

In our study, anesthesia was achieved by intramuscular administration of ketamine hydrochloride (Ketalar, Pfizer, Groton, Custom Tailored Farma, NY, USA) at a dose of 30 mg/kg and xylazine hydrochloride (Rompun; Bayer, Leverkusen, Germany) at a dose of 3 mg/kg.

When necessary, anesthesia was maintained with additional doses (one-third of the first dose) so that the respiratory muscles would contract spontaneously throughout the surgical procedure. In order to prevent possible hypothermia, the surgical procedure was performed in the supine position under a heating lamp. The skin was prepared aseptically and laparotomy was performed in the midline of the abdomen. In order to maintain fluid balance, 10 mL of warm saline was injected into the peritoneal cavity. The intestines were pulled to the left with wet gas to reach IAA. Intravenous administration of 150 U/kg of the anticoagulant heparin (Nevparin, Mustafa Nevzat Pharmaceuticals) was done through the tail vein 2 min before the aortic clamp was clipped. A lower extremity ischemia model was performed by placing a clamp on IAA of rats with a nontraumatic microvascular clamp. The absence of blood flow in the distal of the clamp was confirmed with a hand-held Doppler (HADECO, ES-101 EX model, Hadeco Inc. 2-7-11 Arima, Miyamae-ku, Kawasaki, Japan). The abdominal incision was kept closed to minimize heat and fluid loss. The induced ischemia was observed for 120 min. After occlusion, the abdomen was reopened and the nontraumatic microvascular clamp in IAA was removed and reperfusion was provided for 120 min. IR with aortic clamping was evaluated with the confirmation of the absence of flow in the distal aorta by hand-held Doppler, whereas reperfusion was evaluated with the confirmation of the presence of flow by hand-held Doppler in the distal aorta.

### 2.1. Procurement of samples

At the end of the surgical procedure, after the intracardiac blood of all the rats was collected under anesthesia, the muscle tissues of the lower extremity were quickly removed. The serum was obtained by centrifuging blood samples at 4000 rpm for 5 min and was stored at −80 ºC until the procedure.

The muscle tissues were fixed in 10% formaldehyde solution for immunohistochemical analysis. After fixing, it was washed in tap water. Washed tissues were passed through routine histological follow-up series. The tissues were then embedded in paraffin blocks, and 4–6 micron thick paraffin block sections were cut and collected in polysine slides.

### 2.2. Biochemical analyses

#### 2.2.1. Measurement of total oxidant status (TOS)

Serum TOS levels were measured with TOS assay kit (Rel Assay, Mega Medical Co. Gaziantep, Turkey) [12].

#### 2.2.2. Measurement of serum TRPA1 and TRPC1 levels

Serum TRPA1 (Rat TRPA1 ELISA) kit, SunRed 201-11-6652, China) and TRPC1 (Rat TRPC1 ELISA kit, SunRed 201-11-53005, China) levels in the samples were measured by ELISA.

### 2.3. Immunohistochemical analysis

Paraffin section blocks (4–6 µm thick) were cut and collected in polysine slides. Deparaffinized tissues were processed in graded alcohol solutions and boiled in citrate buffer solution for antigen retrieval at pH 6 in a microwave (750W) for 15 min. After boiling, the tissues were kept at room temperature for about 20 min to cool down before rinsing them with PBS (phosphate buffered saline) (PBS, P4417, Sigma-Aldrich, USA) for 3 x 5 min. Further, the tissues were incubated with hydrogen peroxide block solution (hydrogen peroxide block, TA-125-HP, Lab Vision Corporation, USA) for 5 min to prevent endogenous peroxidase activity. TRPA1 and TRPC1 primary antibodies (anti-TRPA1 antibody, FNab09013, Finetest China and TRPC1 polyclonal antibody, bs-10404R, Biossusa, USA) were diluted by 1:200 after applying Ultra V Block solution (TA-125-UB, Lab Vision Corporation, USA) for 5 min to prevent nonspecific background staining of tissues that were rinsed for 3 x 5 min with PBS. Later, TRPA1 and TRPC1 primary antibodies were incubated in a humid environment for 60 min at room temperature. Tissues were rinsed for 3 x 5 min with PBS after primary antibody incubation, then they were incubated again in secondary antibody (biotinylated goat antipoliyvalent), (antimouse/rabbit IgG), TP-125-BN, Lab Vision Corporation, USA) for 30 min at room temperature in a humid environment. Tissues were rinsed with PBS for 3 x 5 min after secondary antibody application and incubated in streptavidin peroxidase (TS-125-HR, Lab Vision Corporation, USA) for 30 min at room temperature in a humid environment then rinsed with PBS. AEC (3-amino-9-ethyl carbazole) substrate and AEC chromogen (AEC substrate, TA-015 and HAS, AEC chromogen, TA-002-HAC, Lab Vision Corporation, USA) solution was instilled into the tissues and the image signal was taken under the light microscope. Immediately after that, the tissues were rinsed with PBS. Tissues that were counterstained with Mayer’s hematoxylin were rinsed with PBS and distilled water and covered with an appropriate solution (Large Volume Vision Mount, TA-125-UG, Lab Vision Corporation, USA). The prepared sections were examined and photographed under the Leica DM500 microscope (Leica DFC295, Wetzlar-Germany). In the tissues that were prepared for the negative controls PBS was used instead of the primary antibody, other steps were administered routinely.

The histoscore was created based on the prevalence (0.1: < 25%, 0.4: 26%–50%, 0.6: 51%–75%, 0.9: 76%–100%) and severity of immunoreactivity in staining (0: none, +0.5: very low, +1: low, +2: moderate, +3: severe). Histoscore = prevalence x severity.

### 2.4. Statistical analyses

SPSS software v. 22.0 (IBM, Armonk, NY, United States) was used for statistical analyses. Continuous variables were presented as median (minimum–maximum). Kruskal–Wallis test was used for the comparison between more than two groups and significance levels were p < 0.005. Mann–Whitney U test was used for comparison between two groups. In addition, Bonferroni correction was studied and statistical significance level in all tests was 0.01.

## 3. Results

### 3.1. Biochemical findings

#### 3.1.1 .Serum TOS levels

Serum TOS levels of IR group was significantly higher from the remaining groups (p = 0.004). But there was no statistically significant difference for serum TOS levels among the remaining groups. When IR group compared to IR+ILO (p = 0.002), IR+BRL (p = 0.004), IR+ILO+BRL (p = 0.002) groups, a statistically significant decrease was observed in serum TOS levels (Table 1).

**Table 1:** Serum TOS, TRPA1 and TRPC1 levels.

	TOS (µmol/L)Median (min-max)	TRPA1(pg/mL)Median (min-max)	TRPC1(ng/mL)Median (min-max)
SHAM	7.10 (7.02–8.25)	142.94 (142.54–155.28)	3.12 (2.69–3.99)
IR	23.27(11.56–24.07)a	199.10 (168.44–200.67)a	9.89 (6.85–10.23)a
IR+ILO	6.80 (6.75–8.56)b	183.05(168.05–201,05)a	3.12 (3.02–3.45)b
IR+BRL	7.65 (6.35–9.42)b	175.06 (152.73–186.03)a	2.87 (2.15–3.26)b
IR+ILO+BRL	8.13 (6.32–9.23)b	174.65 (173.02–189.65)a	3.15 (2.84–3.25)b
P values of Kruskal– Wallis	0.009	0.021	0.031
P values of Mann–Whitney U	SHAM/IR: 0.004IR/IR+ILO: 0.002IR/IR+BRL: 0.004IR/IR+ILO+BRL: 0.002	SHAM/IR: 0.002IR/IR+ILO: 0.257IR/IR+BRL: 0.024IR/IR+ILO+BRL: 0.024	SHAM/IR: 0.008IR/IR+ILO: 0.008IR/IR+BRL: 0.008IR/IR+ILO+BRL: 0.008

aCompared to sham group.bCompared to IR group (p < 0.01).

#### 3.1.2. Serum TRPA1 levels

In the biochemical analysis of serum TRPA1 levels, there was a statistically significant increase in serum TRPA1 levels in IR group compared to sham (p = 0.002) group (Table 1). IR+ILO (p = 0.257), IR+BRL (p = 0.024), IR+ILO+BRL (p = 0.024) groups were compared with IR group and TRPA1 levels were decreased but these differences were not statistically significant. 

#### 3.1.3. Serum TRPC1 levels

In the biochemical analysis performed to evaluate serum TPRC1 levels, there was a statistically significant increase in IR group compared to sham group (p = 0,008). IR+ILO (p = 0.008), IR+BRL (p = 0.008), and IR+ILO+BRL (p = 0.008) groups were compared to IR group and a statistically significant decrease was observed in TRPC1 levels (Table 1).

### 3.2. Immunohistochemical results

#### 3.2.1.TRPA1 immunoreactivity

**Figure 1:**
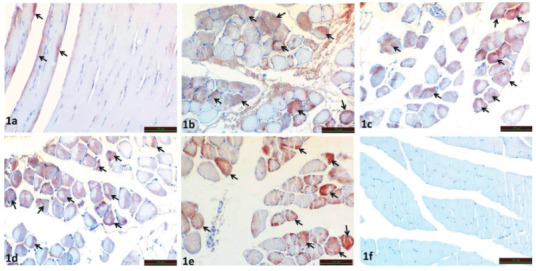
Immunoreactivity of TRPA1 in SHAM (Figure 1a), IR (Figure 1b), IR+ILO (Figure 1c), IR+BRL (Figure 1d), and IR+ILO+BRL (Figure 1e) groups (Black arrow). Negative control (Figure 1f)

Immunohistochemical staining performed under light microscopy revealed TRPA1 immunoreactivity in the myocytes of the muscle tissue (Figure 1).

Compared with sham group (Figure 1a) TRPA1 immunoreactivity was statistically significant increase in IR group (p = 0.005) (Figure 1b) (Table 2). IR+ILO (p = 0.29) (Figure 1c), IR+BRL (p = 0.177) (Figure 1d), and IR+ILO+BRL (p = 0.329) (Figure 1e) groups were compared to IR group. There was no statistically significant difference at TRPA1 immunoreactivity in these group. At the negative control TRPA1 immunoreactivity was not observed (figure 1f) (Table 2). 

**Table 2:** Histoscore for TRPA1 and TRPC1 immunoreactivities.

	TRPA1Median (min-max)	TRPC1Median (min-max)
SHAM	0.20(0.10–0.45)	0.30(0.10–0.45)
IR	0.85(0.60–0.90)a	0.85(0.60–0.90)a
IR+ILO	0.80(0.30–0.90)a	0.40(0.30–0.45)b
IR+BRL	0.70(0.45–0.90)a	0.35(0.20–0.45)b
IR+ILO+BRL	0.60(0.45–0.90)a	0.30(0.20–0.45)b
p values of Kruskal–Wallis	0.001	0.006
p values of Mann–Whitney U	SHAM/IR: 0.005IR/IR+ILO: 0.429IR/IR+BRL: 0.177IR/IR+ILO+BRL: 0.329	SHAM/IR: 0.005IR/IR+ILO: 0.004IR/IR+BRL: 0.004IR/IR+ILO+BRL: 0.004

Values are represented as median, min-max.aCompared to sham group.bCompared to IR group (p < 0.01).

#### 3.2.2. TRPC1 immunoreactivity

**Figure 2:**
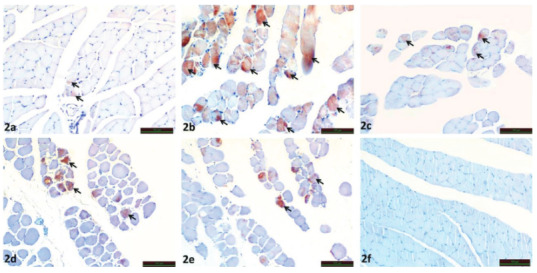
Immunoreactivity of TRPC1 in SHAM (Figure 2a), IR (Figure 2b), IR+ILO (Figure 2c), IR+BRL (Figure 2d), and IR+ILO+BRL (Figure 2e) groups (Black arrow), Negative control (Figure 2f).

For TRPC1 immunoreactivity, immunohistochemical staining performed under light microscopy revealed TRPC1 immunoreactivity in the myocytes of the muscle tissue (black arrow) (Figure 2).

Compared with sham group (Figure 2a), TRPC1 immunoreactivity was statistically significant increase in IR group (p = 0.005) (Figure 2b). IR+ILO (p = 0.004) (Figure 2c) IR+BRL (p = 0.004) (Figure 2d), IR+ILO+BRL (p = 0.004) (Figure 2e) groups were compared to IR group and TRPC1 immunoreactivity was statistically significant decreased. In the negative control there was no TRPC1 immunoreactivity (Figure 2f) (Table 2).

## 4. Discussion

Oxidative stress occurs in many pathophysiological conditions, such as inflammation and reperfusion injuries that occur after ischemia. As a result of oxidative stress, lipid peroxidation products, such as hydrogen peroxide, 4-hydroxynonenal, and prostaglandin J_2 _are expressed [13]. BRL application has shown to reduce the myocardial infarct size and protect the heart against IR damage [14]. Similar to BRL, the antioxidant potential of BRL is known to decrease when these receptors are inhibited [2]. The benefit of ILO administration in preventing the skeletal muscle damage following IR has been demonstrated [3]. In addition, it has been reported that the level of antioxidant enzymes increases during IR damage in rats treated with ILO [4]. Our results show that these two agents reduce elevated serum TOS levels following IR injury. It was observed that there was no difference between the combination of agents given and their separate administration when the effects on serum TOS levels were compared. Consequently, we can infer that they do not promote each other’s effectiveness against oxidative stress.

In cell culture studies, pharmacological activation of TRPA1 channels with ASP 7663 or optovin has been reported to reduce cell death and infarction size in an in vivo rat IR model. Additionally, these agents reduce cell death in isolated ventricular myocytes of rats exposed to IR where TRPA1 expression has been shown [15]. TRPA1 can be activated by active molecules in connection with oxidative stress [13]. Conklin et al. demonstrated that TRPA1 is located in the sarcolemma and intercalated disks in cardiomyocytes, and is a target for lipid peroxidation products [16]. In the same study, it was shown that TRPA1 activated by acrolein cause cell death by increasing calcium influx into cardiomyocytes [16]. In addition, it has been shown that TRPA1 levels increase in solitary nucleus due to lung ischemia [17]. In our study, the strong increase in serum TRPA1 levels following IR damage is in parallel with the previous results. Additionally, it was observed that serum TRPA1 levels in ILO and BRL groups were not similar to those of control group. This showed us that serum TRPA1 levels remained elevated for longer periods, unlike serum TRPC1 levels. It also shows that TRPA1 is more responsive to IR damage and the elevation of TRPA1 may take longer although oxidant molecules are removed from the environment. In addition, the nociceptive effects of ILO are known [18]. The high serum TRPA1 levels after administration of ILO in our study may be due to the nociceptive effect of ILO. The study on mice by So et al. suggest that hypoxia increases the sensitivity of TRPA1 to ROS (reactive oxygen species) and that this plays an important role in painful dysesthesia in peripheral vascular neuropathy [19]. Sasaki et al. showed that the use of antioxidant molecules or TRPA1 blockers may be beneficial in the treatment of allodynia and dysesthesia due to peripheral ischemia [20].

TRPC1 is present in the cell membrane bound to G proteins. When TRPC1 is activated, it allows calcium and alkali cations to enter the cell, causing cell depolarization and increase in intracellular calcium levels [8]. In a cell culture study performed on brain tissue by Xu et al., it was shown that TRPC1 expression decreased during IR injury [21]. In the same study, it was found that the absence of TRPC1 caused a decrease in intracellular calcium, and the infarct area was larger compared to sham group. Calcium or calcium-dependent signaling pathways play a role in a variety of cellular events from cell growth to cell death. One example of calcium’s roles in the skeletal muscles is that for proper functioning of contractile proteins, a well-timed and appropriate amount of calcium is needed. Regulation of calcium flow between the cytosol and sarcoplasmic reticulum in the skeletal muscle cells is vital. In addition, extracellular entry of calcium is also an important step [22]. ROS affect the calcium balance of cells. It has been reported that intracellular calcium decreases because of IR damage disappear with the increase of TRPC1 expression [21]. It also has been suggested that nicotinamid adenin dinucleotid phosphate oxidase 4 (expressions increase in the absence of TRPC1, and this causes an increase in ROS by stimulating nicotinamide adenine dinucleotide phosphate oxidase activity [21]. TRPC1 and caveolin-3 protein levels have been shown to increase in the skeletal muscle from mice with Duchenne muscular dystrophy, and TRPC1 is activated by ROS in an Src kinase-dependent manner [23]. In addition, cell culture experiments have shown that ROS activation of Src significantly increases calcium influx into cells expressing TRPC1 and caveolin-3 [23]. It is known that TRPC1 expression increases in the pulmonary arteries of rats exposed to chronic hypoxia [24]. TRPC1 and TRPC6 have been shown to play important roles in the development of hypoxic pulmonary hypertension in a mouse model that mimics various pathophysiological processes [25]. Additionally, sildenafil and sodium tanshinone IIA sulfonate suppresses TRPC1 and TRPC6 expressions in the treatment of experimental pulmonary hypertension [26,27]. ILO is another drug frequently used in pulmonary hypertension treatment [28]. Our results show that ILO reduces serum TRPC1 levels. Like sildenafil, we suggest that ILO can affect serum TRPC1 levels. In addition, if serum TRPC1 levels in our control group were examined, the decrease in the group in which ILO was given separately supports this phenomenon. 

Although there were no factors limiting the study, both in terms of the number of experimental animals in the study and of the application and termination of the experiment, the lack of polymerase chain reaction and Western blot technique application for protein evaluation is a limitation of the present study and due to experimental design, our results could not be adapted to human beings totally.

In conclusion, In IR group serum TOS, TRPA1 and TRPC1 levels, and tissue TRPA1 and TRPC1 immunoreactivity were statistically significant increase when compared to sham group. In IR+ILO, IR+BRL and IR+ILO+BRL groups serum TRPA1 and tissue TRPA1 immunoreactivity did not change when compared to IR group. Serum TOS and TRPC1 levels, tissue TRPC1 immunoreactivty were statistically significant decreased when compared to IR group. In the pathophysiology of lower extremity ischemia-reperfusion injuries TRPA1 and TRPC1 may have an important role. BRL, in addition to iloprost which is routinely being used clinically and treatment modalities that includes BRL, TRPA1, and TRPC1 need to be investigated in detail for the effects on the ischemia originated diseases.
